# Solving Acoustic Boundary Integral Equations Using High Performance Tile Low-Rank LU Factorization

**DOI:** 10.1007/978-3-030-50743-5_11

**Published:** 2020-05-22

**Authors:** Noha Al-Harthi, Rabab Alomairy, Kadir Akbudak, Rui Chen, Hatem Ltaief, Hakan Bagci, David Keyes

**Affiliations:** 8 School of Computing, University of Utah, Salt Lake City, UT USA; 9 Cray, a Hewlett Packard Enterprise Company, Seattle, WA USA; 10 Helmholtz-Zentrum Dresden-Rossendorf, Dresden, Germany; 11 Extreme Computing Research Center, King Abdullah University of Science and Technology, Thuwal, Saudi Arabia; Extreme Computing Research Center, Computer, Electrical and Mathematical Sciences and Engineering Division, King Abdullah University of Science and Technology, Thuwal, Jeddah, 23955 Saudi Arabia

**Keywords:** Tile low-rank LU-based solver, Boundary Integral Equations, Acoustic scattering, Task-based programming models, Dynamic runtime systems

## Abstract

We design and develop a new high performance implementation of a fast direct LU-based solver using low-rank approximations on massively parallel systems. The LU factorization is the most time-consuming step in solving systems of linear equations in the context of analyzing acoustic scattering from large 3D objects. The matrix equation is obtained by discretizing the boundary integral of the exterior Helmholtz problem using a higher-order Nyström scheme. The main idea is to exploit the inherent data sparsity of the matrix operator by performing local tile-centric approximations while still capturing the most significant information. In particular, the proposed LU-based solver leverages the Tile Low-Rank (TLR) data compression format as implemented in the Hierarchical Computations on Manycore Architectures (HiCMA) library to decrease the complexity of “classical” dense direct solvers from cubic to quadratic order. We taskify the underlying boundary integral kernels to expose fine-grained computations. We then employ the dynamic runtime system StarPU to orchestrate the scheduling of computational tasks on shared and distributed-memory systems. The resulting asynchronous execution permits to compensate for the load imbalance due to the heterogeneous ranks, while mitigating the overhead of data motion. We assess the robustness of our TLR LU-based solver and study the qualitative impact when using different numerical accuracies. The new TLR LU factorization outperforms the state-of-the-art dense factorizations by up to an order of magnitude on various parallel systems, for analysis of scattering from large-scale 3D synthetic and real geometries.

## Introduction

Numerous science and engineering applications require solving large dense linear systems. In particular, the discretization of acoustic Boundary Integral Equations (BIE) using the Nyström method [22, 43] leads to a linear system of equations, where the matrix is dense and non-symmetric. The direct method to solve such a non-symmetric system requires an LU decomposition (or factorization) [30]. LU factorization is an essential operation in linear algebra since it is used in many computational tasks: finding the matrix inverse, computing the matrix determinant, or even ranking the fastest supercomputers with the High Performance LINPACK (HPL) benchmark. As the problem dimensions increase, the cubic and quadratic complexities of the dense LU factorization for arithmetics and memory storage, respectively, make it prohibitively complex.

The matrix operator of the acoustic BIE contains the self-field, near-field, and far-field interactions and, therefore, inherently exhibits a data sparsity structure. Such structure may be exploited using low-rank approximations [31, 37] to attain lower bounds for the arithmetic complexity and memory storage. The achieved accuracy is then controlled with an application-dependent threshold to ensure numerical correctness. The resulting approximated matrix system may then be solved using iterative methods that rely on fast algorithms. For instance, when combined with iterative solvers (e.g., GMRES [49]), the Fast Multipole Method (FMM) [32] may leverage the data sparsity structure and accelerate the matrix-vector multiplication by reducing its complexity from $O(N^2)$ to $O(N\text { log }N)$ or even *O*(*N*).

However, despite their enormous success, iterative methods may still encounter several major bottlenecks when compared to direct solvers. Indeed, iterative methods are often inadequate for ill-conditioned problems which arise when solving a scattering problem near resonant frequencies [2] or when the scatterer exhibits multi-scale geometric features. In contrast, direct methods are stable and are not as sensitive to ill-conditioning. It is only necessary to verify that they are sufficiently well-posed for the required level of accuracy with respect to floating point rounding error. Therefore, when the reliability and predictability of the solver and solution are crucial (e.g., production environments), direct solvers are often preferred. Moreover, iterative methods cannot directly exploit the structure of systems that are altered by a low-rank modification. Direct methods, on the other hand, are particularly effective at handling low-rank perturbations by incrementally updating the existing matrix factors. Last but not least, iterative methods cannot efficiently account for multiple right-hand sides. They often have to start from scratch for each right-hand side (e.g., calculation of monostatic radar cross section) except under certain circumstances (see [28, 29] for economic reuse of Krylov spaces for *nearby* right-hand sides). Direct methods can efficiently work with multiple right-hand sides. As soon as the system matrix has been factorized, direct methods may apply the triangular solves to all right-hand sides at once, resulting in a much lower arithmetic complexity.

In this work, we propose a new high performance implementation of a direct LU-based solver for analyzing acoustic scattering from large 3D objects. The main idea is to exploit the data sparsity of the matrix operator by using the Tile Low-Rank (TLR) data compression format and approximate the off-diagonal tiles. The initial tile-centric compression phase permits to capture the most significant singular values up to an application-dependent accuracy threshold. The LU-based solver can then proceed to using the underlying TLR compressed data structure. We rely on task-based programming models to express the overall TLR LU-based solver into various fine-grained computational tasks operating on tiles. The TLR LU-based solver can actually be translated into a Directed Acyclic Graph (DAG), where nodes correspond to kernels and edges represent the data dependencies. We define an Array of Structure (AoS) of TLR data descriptors to effectively support the data distribution on shared as well as distributed-memory systems. We rely on the dynamic runtime system StarPU [13] to orchestrate the asynchronous executions of the tasks and track their respective data dependencies. We report accuracy results for scattering analysis of large-scale 3D synthetic and real geometries. We show then performance results on several shared-memory systems and compare against the-state-of-the-art dense linear algebra libraries. We further demonstrate the numerical robustness and performance scaling on 1024 nodes of a Cray XC40 dual-socket 16-core Intel Haswell system.

The remainder of the paper is organized as follows. Section 2 describes related work and summarizes our research contributions. Section 3 and 4 recall the background on the formulation and discretization of the acoustic BIE application and dense linear solvers, respectively. Section 5 introduces the task-based TLR LU-based solver algorithm and the corresponding computational kernels. Section 6 provides implementation details on the data distribution and the StarPU dynamic runtime systems. Section 7 highlights the numerical robustness of our TLR LU-based solver using 3D synthetic and real geometry testcases. Section 8 assesses the performance results on various systems and we conclude in Sect. 9.

## Related Work

The application of direct solvers to matrix systems resulting from discretization of 3D problems has traditionally been considered expensive due to the high number of unknowns. However, the synergism between advanced matrix factorization methods, along with modern massively parallel hardware systems have created new opportunities to tackle such challenging problems. Indeed, direct methods have been used together with low-rank matrix representation schemes to reduce the arithmetic complexity. Introduced more than two decades ago, low-rank matrix approximations in the form of hierarchical matrices ($\mathcal {H}$-matrices) [31, 36] represent a new compromise in the literature. Many state-of-the-art data compression formats for $\mathcal {H}$-matrix approximation (e.g., $\mathcal {H}^2$-matrix [16], Hierarchically Semi-Separable (HSS) [7, 26, 48], Block/Tile Low-Rank (BLR / TLR) [5, 9], Hierarchically Off-Diagonal Low-Rank (HODLR) [8, 11]) have been developed to enable the use of finite element method (FEM) and boundary element method (BEM) in analysis of large-scale problems in a broad range of scientific applications. In particular, several $\mathcal {H}$-matrix arithmetics-accelerated schemes have been developed to solve surface integral equations discretized using the Method of Moments (MoM). For instance, direct solvers are coupled with nested and non-nested basis $\mathcal {H}$-matrix compression formats and deployed on shared [24, 40, 47, 50, 51] and distributed-memory systems [12, 34, 35] for large-scale electromagnetic scattering analysis.

**Fig. 1.**
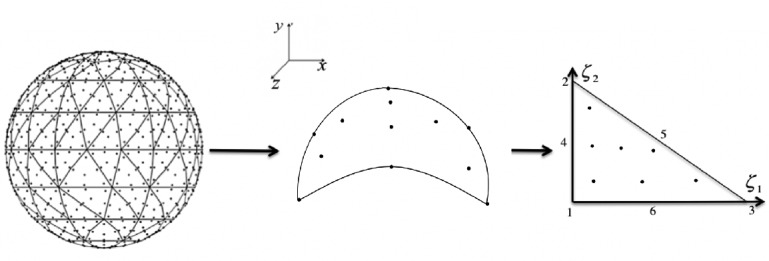
Higher-order geometry modeling: the curvilinear triangular patch in the Cartesian domain and the parent triangle patch in the $(\xi _1, \xi _2)$ domain.

Compared to related work, we design and implement the Tile Low-Rank (TLR) LU-based solver for solving 3D acoustic Boundary Integral Equations (BIE) problems with the Nyström method. In this work, we prefer Nyström over MoM since it allows us to implement a higher-order discretization in a more straightforward way, without loosing generality. By adopting TLR, i.e., a flat tree data compression format, we trade off optimality with user productivity to reduce the deployment effort on massively parallel systems. Such approach has already demonstrated its effectiveness in solving large-scale scientific problems [6, 23]. In fact, TLR may be considered as one step toward bridging the complexity gap (i.e., arithmetic and memory) between flat and hierarchical low-rank matrix formats [10]. Moreover, our systematic approach relies on asynchronous task scheduling using the dynamic runtime system StarPU to cope with the load imbalance issue. We apply our new solver to the analysis of scattering from 3D synthetic and real geometries, evaluate the numerical robustness and assess the performance results on various parallel systems.

## The Acoustic Boundary Integral Equation Application

**Problem Definition.** For an acoustically rigid scatterer, the Helmholtz Boundary Integral Equation (BIE) in unknown pressure field $P(\mathbf {r})$ reads:

1 $$\begin{aligned} \frac{1}{2}P(\mathbf {r})-\int _S P(\mathbf {r'}){\partial _{n'} G(\mathbf {r,r'})}dS'=P^{\text {inc}}(\mathbf {r}),\, \mathbf {r}\in S, \end{aligned}$$

where *S* is the surface of the scatterer, “$\partial _{n'}$” denotes the partial derivative in the direction of the surface normal unit vector $\mathbf {\hat{n}}(\mathbf {r}')$, $G(\mathbf {r,r}')={e^{jk|\mathbf {r-r'}|}}/{(4\pi |\mathbf {r-r'}|)}$ is the 3D scalar Green function of the unbounded domain where *S* resides in, $P^{\text {inc}}(\mathbf {r})$ is the pressure field, and $\mathbf {r'}$ and $\mathbf {r}$ are source and observer points, respectively.

**Higher-Order Discretization Using Nyström Method.** To facilitate the numerical solution of Eq. (1), first, *S* is divided into a mesh of *N* curvilinear triangular patches, as seen in Fig. 1. A higher-order Nyström method [45] is then used on this mesh to discretize Eq. (1). To this end, the scalar version of the vector interpolation functions that have been introduced in [42] is used to expand the unknown $P(\mathbf {r})$. This function requires *M* number of interpolation points to be defined on each patch. Inserting this expansion into Eq. (1) and testing the resulting equation at these interpolation points of each patch yield a linear system of equations $\bar{A} \bar{P}= \bar{P}^\mathrm{inc},$ where $\bar{A}$ is a matrix of dimension $NM \times NM$, $\bar{P}^\mathrm{inc}$ and $\bar{P}$ are vectors of dimension *NM* storing samples of test incident field $P^\mathrm{inc}(\mathbf {r})$, and (unknown) the pressure field $P(\mathbf {r})$ at the interpolation points. Their entries are given by:

2 $$\begin{aligned} \bar{A}_{ef,qj}&= \frac{1}{2}\delta _{ef,qj}-\int _{S_q} \partial _{n'} G(\mathbf {r}_{qj},\mathbf {r'})L_{qj}(\mathbf {r'})ds', \nonumber \\ \bar{P}^\mathrm{inc}_{ef}&=P^\mathrm{inc}(\mathbf {r}_{ef}), \nonumber \\ \bar{P}_{qj}&=P(\mathbf {r}_{qj}), \end{aligned}$$

for $e,q=1,\ldots , N$, and $f,j=1,\ldots , M$. Here, $\delta _{ef,qj}=1$ for $ef=qj$ and zero otherwise, $\mathbf {r}_{ef}$ is the testing (interpolation) point *e* on patch *f*, $\mathbf {r}_{qj}$ is the source (interpolation) point *q* on patch *j*, $S_q$ is the surface of patch *q*, and $L_{qj}(\mathbf {r})$ is the interpolation function associated with point $\mathbf {r}_{qj}$.

The surface integral in Eq. (2) is evaluated numerically after it is "mapped" onto a unit flat right-angle triangle (see Fig. 1). Furthermore, the computation of this integral calls for a singularity treatment scheme when $e=q$. Several approaches have been proposed in the literature (e.g., Duffy transformation [27], singularity subtraction technique (SST) [41], and polar coordinate transformation [33]). In this work, we use the polar coordinate transformation (PCT) based on the improved Guiggiani’s method [18].

It should also be noted here that the acoustic BIE formulation used in this work suffers from the internal resonance problem, i.e., Eq. (1) has a null space at the frequencies that coincide with cavity modes/resonances of *S* [15]. In other words, the pressure field solution on the surface of the scatterer does not generate any scattered/radiated fields. These resonance frequencies depend on the shape of the scatterer. The condition number of the matrix resulting from the discretization of the integral equation increases as the excitation frequency approaches any one of these resonance frequencies. Unless the two frequencies exactly coincide, the matrix system might still be solved but often iterative methods do not converge or require too many iterations to be considered efficient. For such cases, direct methods produce the solution more efficiently assuming the matrix is well-posed enough for the required level of accuracy with respect to floating point rounding error. Note that for the examples considered in the paper, the excitation frequency is sufficiently away from the resonance frequencies. The Burton-Miller formulation [20] can alleviate this problem of non-uniqueness. However, the discretization of this formulation requires the use of more complex singularity treatment techniques (particularly for higher-order discretizations) as the order of singularity increases. An accurate numerical result can still be achieved, even with the existence of the internal resonance problem, if the frequency does not coincide exactly with the resonance frequencies [25].

Once the linear system is built, the non-symmetric matrix $\bar{A}$ in double complex precision arithmetic is diagonally dominant due to the “self-patch interactions”. The LU factorization may represent the adequate choice for direct solving the dense linear system, without the pivoting mechanism. Moreover, its off-diagonal blocks are usually data sparse and may be subject to low-rank approximations. Last but not least, the matrix $\bar{A}$ may need a proper row/column ordering of the patch indices [44] to decouple the “near-patch interactions” from “far-patch interactions” so that the compression phase may be further optimized.

## The State-of-The-Art Dense LU-Based Solvers

LAPACK and ScaLAPACK are the *de facto* libraries for performing dense linear algebra operations. However, the algorithmic paradigm has shifted from block (i.e., coarse granularity) to tile (fine granularity) algorithms in response to manycore hardware evolution. Block algorithms emphasize efficient use of deep memory hierarchies, whereas tile algorithms focus on achieving a high level of concurrency.

**Block LU Algorithm.** The LU factorization provided in LAPACK and ScaLAPACK software packages is implemented as a high-level algorithm built on top of the Basic Linear Algebra Subprograms (BLAS) for LAPACK and the Basic Linear Algebra Communication Subprograms (BLACS) for ScaLAPACK. Conceptually, the matrix is divided into blocks of columns, commonly named panels, on which a partial pivoting scheme may be applied. The factorization then proceeds by an update of the trailing submatrix. This panel-update sequence continues until all panels are factorized. Block algorithms leverage the Level-3 BLAS matrix-matrix multiplication GEMM, resulting into a superior data reuse which is required to run efficiently on cache-based computer architectures. However, in-between synchronization points are required, due to the fork-join paradigm. The parallelism occurs only within each update phase and is expressed at the BLAS and BLACS levels, which eventually degrades the performance [3].
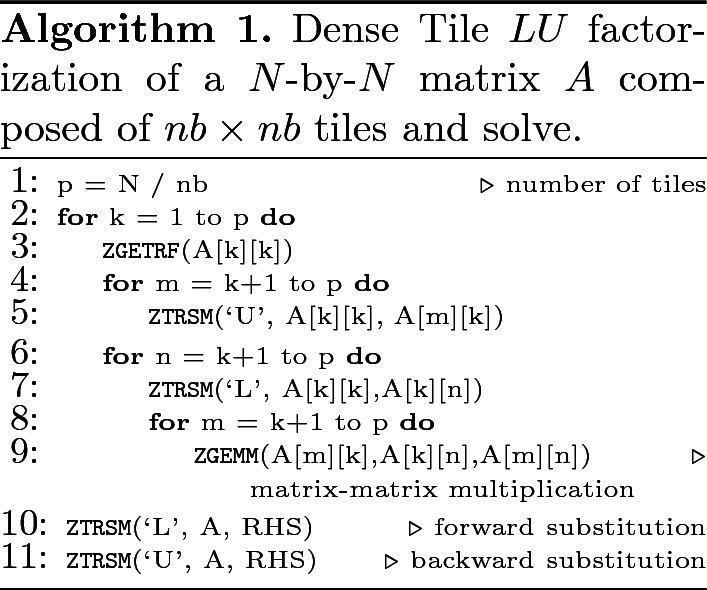


**Fig. 2.**
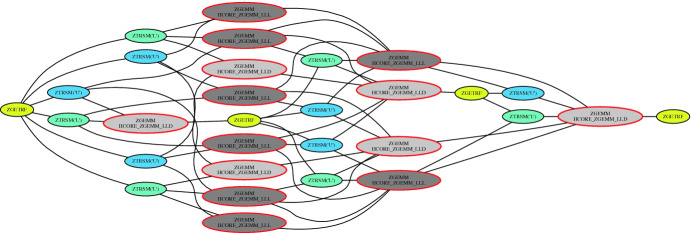
Single DAG for the tile dense and tile low-rank LU factorization for a 4-by-4 matrix. Yellow nodes represent ZGETRF operations, blue and cyan nodes represent upper and lower ZTRSM operations, resp., and dark and light gray nodes represent ZGEMM and HCORE_ZGEMM_XXX operations, respectively. (Color figure online)

**Tile LU Algorithm.** To alleviate the synchronization bottleneck seen in block algorithms, the dense linear algebra community introduced a decade ago a redesign of matrix computation algorithms, named tile algorithms, using task-based programming models. The idea consists in splitting the matrix into tiles, on which elements are contiguous in memory for better cache usage. The panel factorization and the update of the trailing submatrix may now be represented into successive fine-grained computational tasks operating on tiles. The fine granularity tasks weaken the synchronization points seen in block algorithms and create opportunities for asynchronous execution. The sequential tile algorithms can then be presented by a Directed Acyclic Graphs (DAG), where nodes and edges represent the computational tasks and the dependencies among the tasks, respectively. The key idea is to bring the parallelism to the fore by scheduling the DAG’s sequential tasks using a dynamic runtime system. The runtime system is then in charge of orchestrating the tasks across the underlying shared and distributed-memory resources, while ensuring data dependencies are not violated. The performance advantages of tile over block algorithms have been discussed in the literature [4, 17, 21, 46]. Algorithm 1 shows the pseudo-code of the dense tile LU-based solver in double complex precision arithmetic, involving three kernels, i.e., ZGETRF, ZTRSM, and ZGEMM, performing the LU factorization of the diagonal tile, the triangular matrix solve and the matrix-matrix multiplication, respectively. The corresponding DAG of the tile LU factorization for a 4-by-4 tile matrix is drawn in Fig. 2: the DAG width shows the critical path and the height exposes the concurrency.

## The Tile Low-Rank LU-Based Solver Algorithm

**The Tile Low-Rank Compression Format.** Following the principle of the dense tile algorithms, the Tile Low-Rank (TLR) algorithm exploits the data sparsity of the off-diagonal tiles [5, 6]. The initial phase is to approximate each of the off-diagonal tiles of size *nb* using the fast randomized singular value decomposition [39], while capturing only the most significant *k* singular values and their associated singular vectors. The rank *k* depends on the user-defined accuracy threshold. The diagonal tiles are typically full rank and may not be approximated. Then, each of the off-diagonal tiles (*i*, *j*) can be represented by the product of two rectangular matrices $U_{ij}$ and $V_{ij}$ of size $nb \times k$. Once the tile-centric compression phase ends, the TLR LU-based solver phase can then carry on with the TLR matrix computation. 
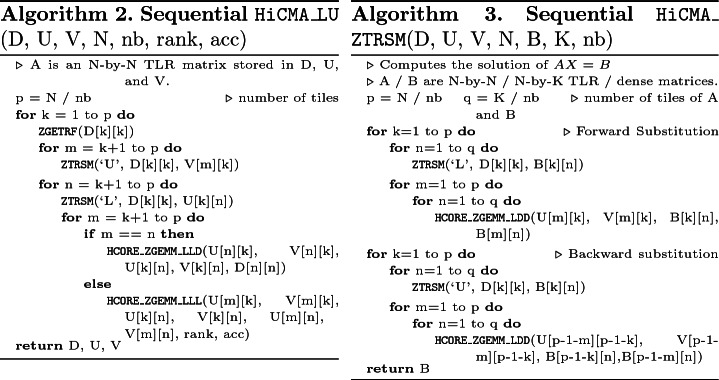


**Description of the Numerical Kernels.** The TLR LU-based solver algorithm requires new computational kernels. Compared to the sequential dense LU algorithm, the sequential TLR LU algorithm is quite similar, except that it necessitates a new matrix-matrix multiplication kernel that takes into account the data format (i.e., dense or TLR) of each operand A, B, and C. The new HCORE_ZGEMM_XXX has three variants to fully support the TLR LU-based solver algorithm: (1) HCORE_ZGEMM_LLL performing C = C+A*B, where A,B,C are TLR, (2) HCORE_ZGEMM_LLD performing C = C+A*B, where A, B are TLR; C is dense, and (3) HCORE_ZGEMM_LDD performing C = C+A*B, where A is TLR; B, C are dense (used in solve part). Algorithms 2 and 3 highlight the pseudo-codes of the sequential TLR LU factorization and its corresponding solver, respectively. The diagonal tiles need a special treatment for the matrix-matrix multiplication due to their dense data structure (i.e., HCORE_ZGEMM_LLD). The corresponding DAG of the TLR LU factorization for a 4-by-4 tile matrix is essentially the same as the tile dense LU, as shown in Fig. 2. However, the red circle tasks highlight the difference when performing the matrix-matrix multiplication kernel with dense tile LU (i.e., ZGEMM) or TLR LU (i.e, the HCORE_ZGEMM_XXX variants).

The tile size *nb* is a tunable parameter and has a significant effect on the overall performance as it trades off optimality and parallel performance [5, 6, 23]. The fixed accuracy allows for an application-dependent level of approximation for the off-diagonal tiles. However, this may engender variable ranks across off-diagonal tiles, which may cause load balancing issues. It is then paramount to rely on a dynamic runtime system to mitigate the load balancing issues, while maintaining high occupancy on the underlying hardware resources.

## Implementation Details

**Array of TLR Structures.** We develop the new TLR LU factorization in the context of the Hierarchical Computations on Manycore Architectures (HiCMA [1] ) software library. We have extended HiCMA to support non-symmetric matrix computations, and given the BIE problems, we have also provided support for double complex precision arithmetic. HiCMA inherently provides a data descriptor for TLR using a 2D block cyclic data distributions, similar to the ScaLAPACK descriptor [5]. The TLR data descriptor specifies how the data should be distributed among processing units. To reduce the memory footprint, we have changed the existing Structure of TLR Arrays (SoA) for the data descriptor to an Array of TLR Structures (AoS). This allows us to allocate each logical tile using their respective sizes that depend on the rank *k*. This flexibility in handling the rank disparities is critical, especially when moving data in distributed-memory system environments. We also generate on-the-fly each dense tile individually using reusable buffers, before compressing them using the randomized SVD [39]. We initially allocate as many buffers as the number of processing units so that the matrix does not need to be wholly alive at any given time. Although TLR does not provide linear complexity, this format has enabled to solve challenging problems with a high number of unknowns on massively parallel distributed-memory system [6, 23]. TLR adopts a flattened algorithmic design to further increase task parallelism in lieu of the plain recursive approach usually adopted in $\mathcal {H}$-matrix libraries [38]. TLR is actually a step toward reducing the complexity gap (i.e., arithmetic and memory) between flat and hierarchical low-rank matrix formats [10]. HiCMA relies on the dynamic runtime system StarPU to run in parallel, which is explained in the next section.
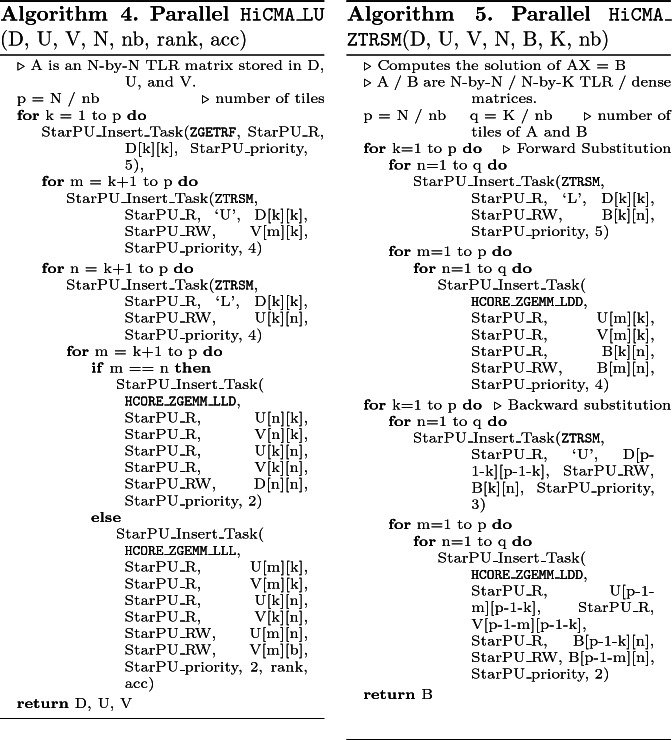


**The StarPU Task-Based Runtime Systems.** StarPU [13] is the standard dynamic runtime system for HiCMA. It handles the execution of generic task graphs, which results from the Sequential Task Flow (STF) programming model. Herein, the tasks are exposed to the runtime with hints on the data directions (i.e., StarPU_R, StarPU_W, and StarPU_RW). Then, the StarPU runtime system starts to dynamically schedule the given tasks asynchronously based on the given data directions. The StarPU runtime system abstracts the complexity of the underlying hardware and improve user productivity. StarPU supports shared and distributed-memory systems (possibly equipped with GPUs). StarPU plays an even more major role when dealing with scattering analysis from 3D acoustic BIE problems than 2D Gaussian process, as seen in [5, 6]. The large rank discrepancy between off-diagonal tiles is more severe in the herein studied applications. This creates load imbalance situations where resource may become idle. Thanks to the fine-grained computational tasks, StarPU can perform asynchronous executions by exploiting algorithmic lookahead to mitigate theses overheads and maximize hardware occupancy. Lookahead is achieved at runtime by using the task priority and the task window size features from StarPU. Basically, the task window size governs the number of queued tasks. The more tasks are queued, the more opportunities to create lookahead opportunities. Once the tasks shift to a ready state (i.e., tasks for which data dependencies are satisfied), they are executed following the order of their priorities. Algorithms 4 and 5 show the pseudo-codes of the parallel TLR LU factorization and its corresponding solver. The API StarPU_Insert_Task queues the tasks with the data directions for each operand and eventually executes them, as soon as their data dependencies are satisfied. One of the interesting things to notice is the user productivity achieved when moving from sequential (i.e., Algorithms 2 and 3) to parallel TLR LU-based solver (i.e., Algorithms 4 and 5).

## Numerical Accuracy

**Fig. 3.**
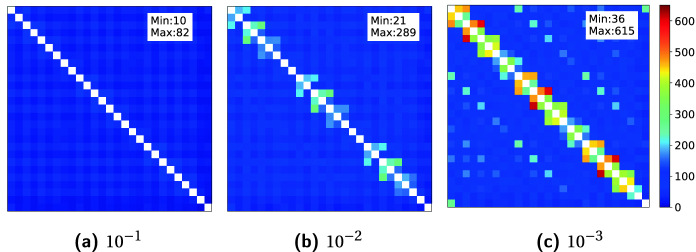
The rank distribution of initial matrices obtained by compression for different accuracy thresholds. The matrix size is 91368-by-91368 and tile size $nb=3384$. There are 27 tiles in each dimension. The colors show the ranks of tiles, i.e., red tones denote larger ranks, whereas blue tones denote smaller ranks. For smaller thresholds, both average and maximum ranks become larger. (Color figure online)

We assess the numerical accuracy and robustness of the HiCMA TLR LU-based solver. Figure 3 pictures the rank distribution of the initial matrix obtained after compression and presents the impact of using different accuracy thresholds on the matrix structure. The matrix size is 91368-by-91368 and tile size $nb=3384$. There are 27 tiles in each dimension. The colors show the ranks of tiles, i.e., white tiles show the dense diagonal with full rank, red tones denote tiles with larger ranks, whereas blue tones denote tiles with smaller ranks. The highest ranks are located on tiles around the diagonal, which contains the near-field strong interactions. The smallest ranks are located on the off-diagonal tiles, which typically represent the far-field weak interactions. Furthermore, as the thresholds decrease, both average and maximum ranks become larger since the truncation step removes less singular values.

**Fig. 4.**
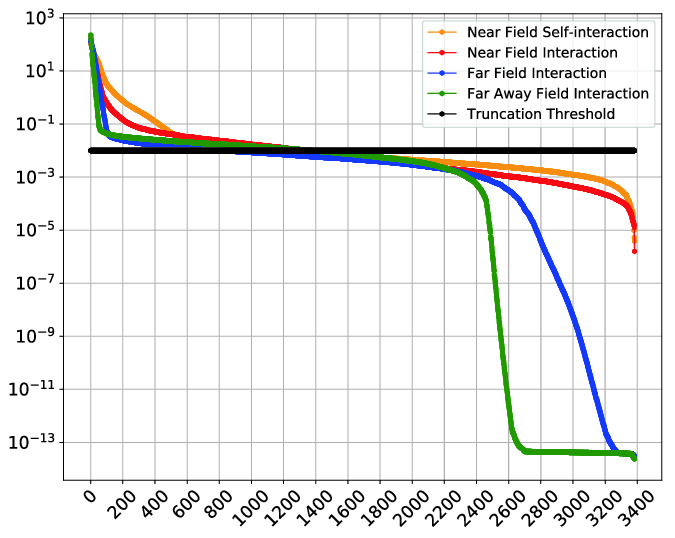
Singular Values Decay for blocks (13,12), (15,11), (20,5) , and (27,1), respectively, of matrix size 91368-by-91368 and tile size is 3384

Singular value decay for specific tiles is depicted on Fig. 4. The tile location is chosen according to the distance from source point. We select tile on sub-diagonal which is close to self-interaction field, tile on the near field interaction, tile on the far interaction area, and last tile is on the farthest point. We can notice that when moving away from the source point the singular values decayed significantly compare to the near field interaction tile.

**Fig. 5.**
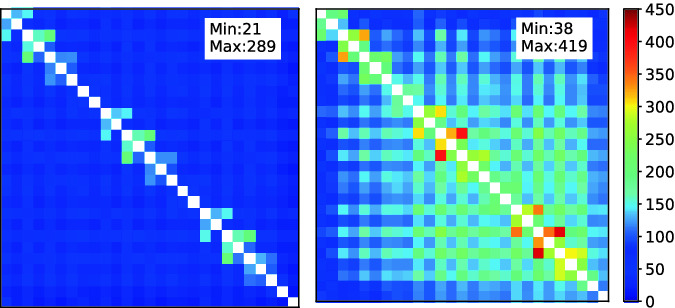
The rank distribution before (on the left) and after (on the right) LU factorization. The matrix size is 91368-by-91368 and tile size is 3384, the accuracy threshold is $10^{-2}$. White color is used to represent dense tiles. The ranks become larger after factorization. (Color figure online)

Figure 5 shows the rank distribution before and after HiCMA TLR LU factorization for the matrix size of 91368-by-91368. The tile size is 3384 where 27 tiles in each dimension, and the accuracy threshold is $10^{-3}$. It is interesting to see that the rank growth has been limited throughout the factorization.

**Fig. 6.**
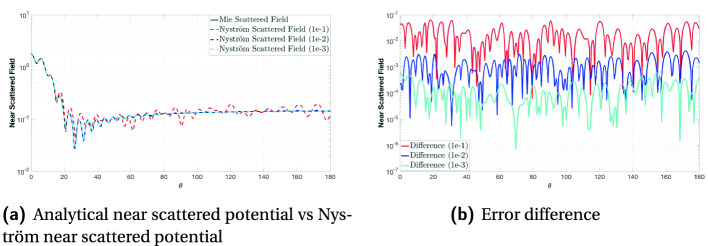
Comparison of the near scattered potential computed on a circle with radius $a=4$ m for $\phi ={0}^\circ$ and ${0}^\circ<\theta <{180}^\circ$ using the numerical solution (obtained by the HiCMA LU solver for accuracy $1e-1$, $1e-2$ and $1e-3$) and the analytical solution, obtained using Mie series. The matrix size is 91368-by-91368 and tile size is 3384.

To validate and demonstrate the accuracy of the solver, we consider scattering by a rigid sphere, so that the scattering fields can be easily verified and tested by comparing them with the analytical solutions (Mie Series Solution). Figure 6 compares the near scattered fields with the Mie scattered fields on a sphere with a radius $a=1$ m, and is discretized into $N=15228$ high-order curvilinear iso-parametric quadrilateral elements and resulting in 91368 number of unknowns. The incident wave is a uniform plane wave with a frequency $f=1978$ Hz, and the propagation medium is air where the standard speed of sound in air is $c=340.29 m/s$. The solution is obtained using the HiCMA TLR LU-based solver for three different fixed accuracies, i.e., $1e-1$, $1e-2$ and $1e-3$. Then, as a post-processing step, we calculate the potential on a circle with a radius of 4m ($\theta \in [{0}^\circ ;{180}^\circ ]; \phi = {0}^\circ$). Figures 6a and 6b plot the scattered potential amplitude and its difference from that computed using a Mie series code versus $\theta$ of this circle. We can see the error decreases as the accuracy increases, which demonstrates the correct implementation of the solver. While $1e-1$ degrades the numerical solution, an accuracy threshold of $1e-2$ is typically satisfactory for the application requirement. With the accuracy threshold of $1e-3$, the solver is in an “over-accurate” state, which results in an unnecessary computational load.

**Fig. 7.**
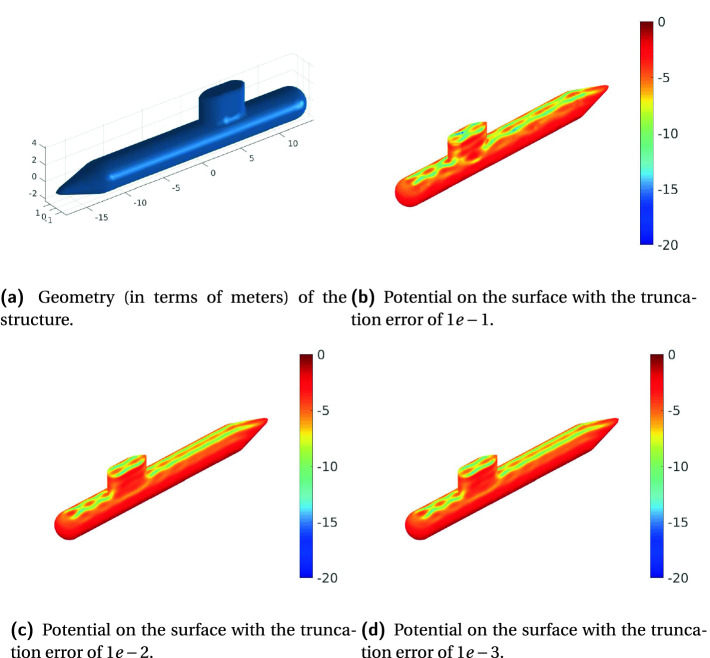
Application of HiCMA TLR LU-based solver to the analysis of acoustic scattering from a submarine.

Next, to show the applicability of the solver, we consider scattering from a more complex realistic submarine geometry, as shown in Fig. 7. The submarine is contained within a box of dimensions $32.1 \times 3.6 \times 6.5$ meters, as shown in Fig. 7a. Its surface is discretized into 10818 second-order curvilinear triangular patches resulting in having 64908 unknowns. The submarine is illuminated by an x-propagating plane wave with a frequency of 1366 Hz. Figures 7b, 7c, and 7d show the pressure fields (in dB) induced on the surface of the submarine computed by the HiCMA TLR LU-based solver for three different truncation errors, 1e-1, 1e-2 and 1e-3, respectively. Based on conclusion from the previous set of results for the sphere and since there is no significant difference between Figs. 7c and 7d, we believe the solution converged and thus a 1e-2 accuracy for this example is enough.

## Performance Results

**Environment Settings.** The experiments are carried on several shared and distributed-memory systems, as illustrated in Table 1. All computations are performed using double complex precision arithmetic and the presented results are an average of three consistent executions. HiCMA and StarPU (v1.2.6) are built by GCC (v5.5.0) on Intel and AMD shared-memory systems and by ARM Allinea Studio (v19.2) on the ARM system. For BLAS and LAPACK implementations, MKL (v2018) is used on the Intel systems, OpenBLAS (v0.2.20) on the AMD system, and ArmPL (v19.2) on the ARM system. On the distributed-memory system, HiCMA and StarPU (v1.2.6) have been compiled with Intel compiler suite (v18.0.1.163) and link against Cray MPICH.

**Fig. 8.**
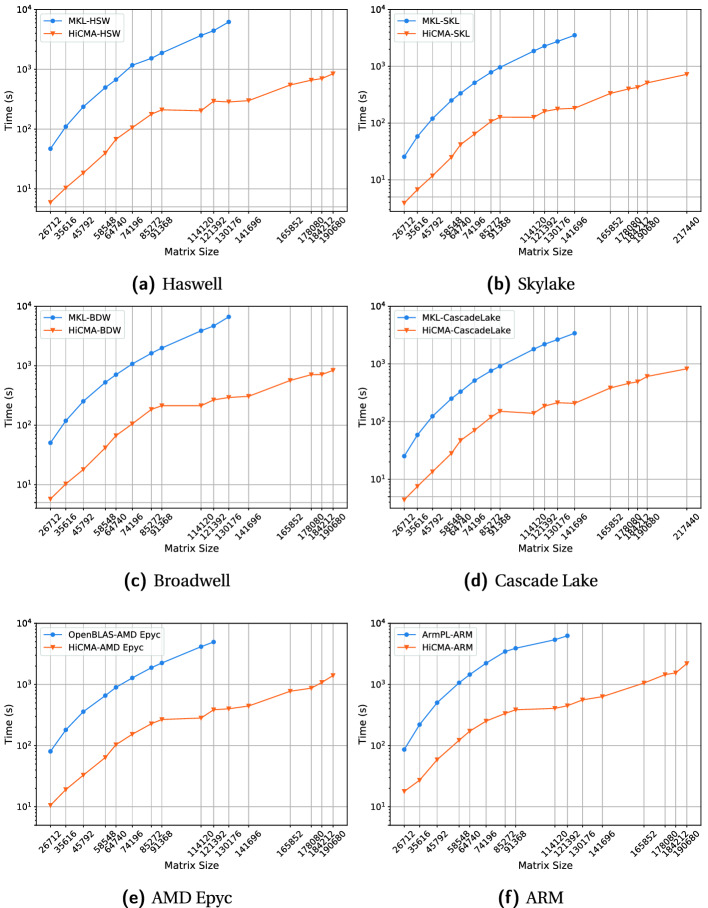
Performance of HiCMA TLR LU versus vendor optimized dense LU on Intel systems (Haswell, Skylake, Broadwell, Cascade Lake), AMD Epyc, and ARM shared-memory systems.

**Performance Comparisons.** Fig. 8 presents the performance comparisons in time of vendor optimized dense LU factorization versus HiCMA TLR LU factorization. HiCMA TLR LU is up to an order of magnitude faster than the vendor optimized dense LU factorization across all systems. This figure demonstrates the portability of HiCMA TLR LU factorization. The times shown do not include the the matrix generation for both HiCMA TLR LU and dense LU, nor the compression phase for HiCMA TLR LU only.

**Table 1.** Hardware specifications.

	*Shaheen-2*	Broadwell(BDW)	Haswell(HSW)	Skylake (SKL)	Cascade Lake	AMD	ARM
Family	E5V3	E5V4	E5	Scalable	Scalable	EPYC	Marvell
Model	2698	2680	2699	2698	6248	7601	ThunderX2
Node(s)	6144	1	1	1	1	1	1
Socket(s)	2	2	2	2	2	2	2
Cores	32	28	36	40	40	64	64
GHz	2.60	2.40	2.30	2.40	2.50	2.2	2.5
DDR4 (GB)	128	256	256	370	370	256	256
L3 cache (MB)	40	35	35	27.5	28	64	32

**Fig. 9.**
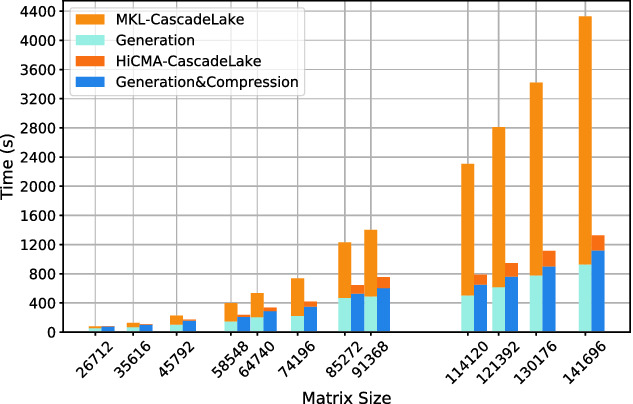
Time breakdown comparing HiCMA Generation, Compression, and Computation with MKL Generation, and Computation on Cascade Lake shared-memory systems.

**Time Breakdown.** Fig. 9 shows the time spent in the generation and compression phases for the HiCMA TLR LU and dense LU on the Cascade Lake system for various matrix sizes. The time for the compression phase is negligible compared to the overall time to solution. The time for generation may be improved using Adaptive Cross Approximation technique [14] but this is beyond the scope of the paper.

**Fig. 10.**
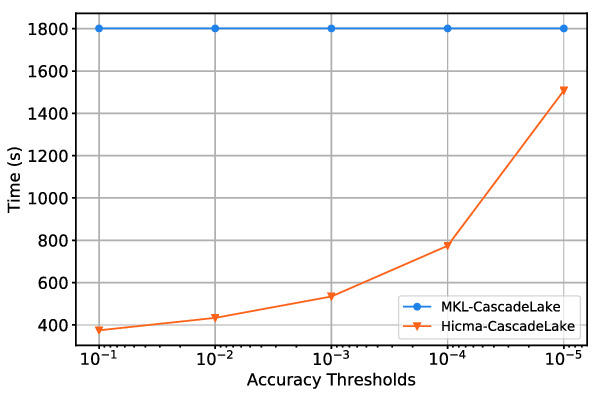
Runtime HiCMA TLR LU factorization on Cascade Lake system for different accuracy thresholds. The matrix size is 114120-by-114120 and tile size $nb=1902$.

**Accuracy Impact on the Performance.** Fig. 10 shows the accuracy impact on the overall performance. As we increase the accuracy threshold, the time to solution increases steadily due to larger ranks *k* captured on each tile. The HiCMA TLR LU may actually becomes slower than the vendor optimized dense LU due to the expensive recompression step once $k>nb/2$.

**Fig. 11.**
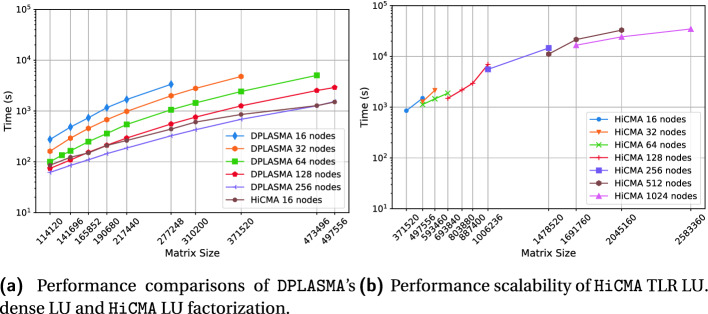
Performance assessment on distributed-memory environment.

**Performance on Distributed-Memory System.** Fig. 11a shows the performance of the dense LU factorization (i.e., the zgetrf_nopiv double complex LU routine without pivoting) from DPLASMA v2.0 [17] on up to 256 nodes and compares it against HiCMA LU factorization on 16 nodes only. Thanks to low-rank approximations, HiCMA TLR LU outperforms DPLASMA’s dense LU across a range of matrix sizes, and up to an order of magnitude when using the same number of nodes. Figure 11b shows the performance scalability of HiCMA TLR LU on up to 1024 nodes with 2.5M unknowns.

**Fig. 12.**
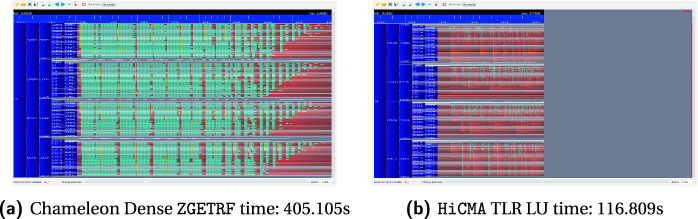
Execution traces on four nodes with a matrix size of 74K. (Color figure online)

**Traces.** Fig. 12 presents the execution trace of tile dense LU factorization as implemented in Chameleon and HiCMA TLR LU on four nodes with a matrix size of 74K. Each core of the four nodes has it timeline represented along the x axis. The blue colors corresponds to cores busy working, while the red colors show when the cores are idle. The gray area for the HiCMA trace does not record any activities since the code has already finished. While it is true that HiCMA TLR LU is much faster than tile dense LU factorization from Chameleon, we believe there is still room for improvement. Some of the off-diagonal tiles may have higher ranks, as seen in Fig. 5. Further runtime optimizations may be required to execute tasks along the critical path with higher priorities. Also, as seen in previous work [23], a hybrid data distribution combining 1D cyclic for the dense diagonal tiles and 2D cyclic for the off-diagonal tiles may result in a better load balancing.

## Summary and Future Work

We present a new high performance implementation based on the Tile Low-Rank (TLR) LU factorization, which exploits the data sparsity of the matrix operator, in the context of scattering analysis from acoustic 3D boundary integral equations. Our high performance TLR LU factorization relies on task-based programming models associated with the StarPU dynamic runtime system. We extend and plan to integrate this new TLR LU into the existing HiCMA TLR software library. This synergistic software solution enables asynchronous executions of the TLR LU factorization, while mitigating the data motion overhead. We compare the obtained performance results of our TLR LU factorization against the state-of-the-art dense factorizations on several shared and distributed-memory systems. We achieve up to an order of magnitude performance speedup. We are able to solve the scattering analysis from 3D acoustic BIE problem with up to 2.5M unknowns in double complex precision arithmetics. Although our algorithmic and software solutions rely on StarPU, we would like to investigate the PaRSEC dynamic runtime system, which uses a domain specific language to target extreme scale performance [23]. We would like also to assess more complex geometries [19] and to provide support for GPU hardware accelerators. Another direction may be the study of our fast direct solver in the context of a monostatic radar cross-section scattering problem involving multiple right-hand sides. Last but not least, we plan to release the new TLR LU-based solver into the HiCMA open-source library.
